# Association of leisure time physical activity and NMR-detected circulating amino acids in peripubertal girls: A 7.5-year longitudinal study

**DOI:** 10.1038/s41598-017-14116-2

**Published:** 2017-10-25

**Authors:** Xiaobo Zhang, Petri Wiklund, Na Wu, Yifan Yang, Haihui Zhuang, Sulin Cheng

**Affiliations:** 1 School of Life Sciences and Biotechnology, Shanghai Jiao Tong University, Shanghai, China; 2 Center for Life Course Health Research, University of Oulu, Oulu, Finland; 3 Department of Epidemiology and Biostatistics, School of Public Health, Imperial College London, London, UK; 4 Faculty of Sport and Health Sciences, University of Jyväskylä, Jyväskylä, Finland

## Abstract

This study investigated the longitudinal associations of physical activity and circulating amino acids concentration in peripubertal girls. Three hundred ninety-six Finnish girls participated in the longitudinal study from childhood (mean age 11.2 years) to early adulthood (mean age 18.2 years). Circulating amino acids were assessed by nuclear magnetic resonance spectroscopy. LTPA was assessed by self-administered questionnaire. We found that isoleucine, leucine and tyrosine levels were significantly higher in individuals with lower LTPA than their peers at age 11 (p < 0.05 for all), independent of BMI. In addition, isoleucine and leucine levels increased significantly (~15%) from childhood to early adulthood among the individuals with consistently low LTPA (p < 0.05 for both), while among the individuals with consistently high LTPA the level of these amino acids remained virtually unchanged. In conclusion, high level of physical activity is associated lower serum isoleucine and leucine in peripubertal girls, independent of BMI, which may serve as a mechanistic link between high level of physical activity in childhood and its health benefits later in life. Further studies in peripubertal boys are needed to assess whether associations between physical activity and circulating amino acids in children adolescents are sex-specific.

## Introduction

An active lifestyle elicits many health benefits. Studies have shown that high level of habitual physical activity is associated with reduced risk of cardio-metabolic disease, type 2 diabetes and all-cause mortality^1^. These effects are partly mediated by a variety of metabolic adaptations to physical activity; it is well-known that exercise increases mobilization of lipids from adipose tissue towards skeletal muscle for fatty acid oxidation^2^, which is why exercise has the capacity to induce loss of fat mass and therefore weight loss^3^. In addition to the ability to increase fatty acid oxidation, exercise appears to also promote oxidation of amino acids. Studies have shown that long-term leisure-time physical activity is associated with increased branched-chain amino acid catabolism in the skeletal muscle and adipose tissue^4^, and low circulating branched-chain amino acid concentrations^5^. It is believed that branched-chain amino acids contribute to energy metabolism during exercise as energy sources and substrates to expand the pool of citric acid–cycle intermediates, thus allowing more efficient mitochondrial energy production from lipids and hereby better metabolic health^6^. These findings are intriguing, given that elevated levels of branched-chain amino acids have long been associated with obesity, insulin resistance and cardiovascular disease^7–10^. More studies are therefore needed for better understanding how habitual physical activity affects amino acid metabolism.

One gap in the literature is the lack of data on the relationship between physical activity and circulating amino acid profiles in peripubertal girls in whom somatic growth during puberty and adolescence, as well as changes in proteolysis and protein oxidation is expected to affect circulating amino acid levels^11^. In the present study, we investigated the longitudinal associations of leisure-time physical activity (LTPA) and circulating amino acids concentrations in peripubertal girls transiting from pre-puberty to early adulthood.

## Results

### Changes in patterns of amino acids, physical activity during pubertal growth

**Figure 1:**
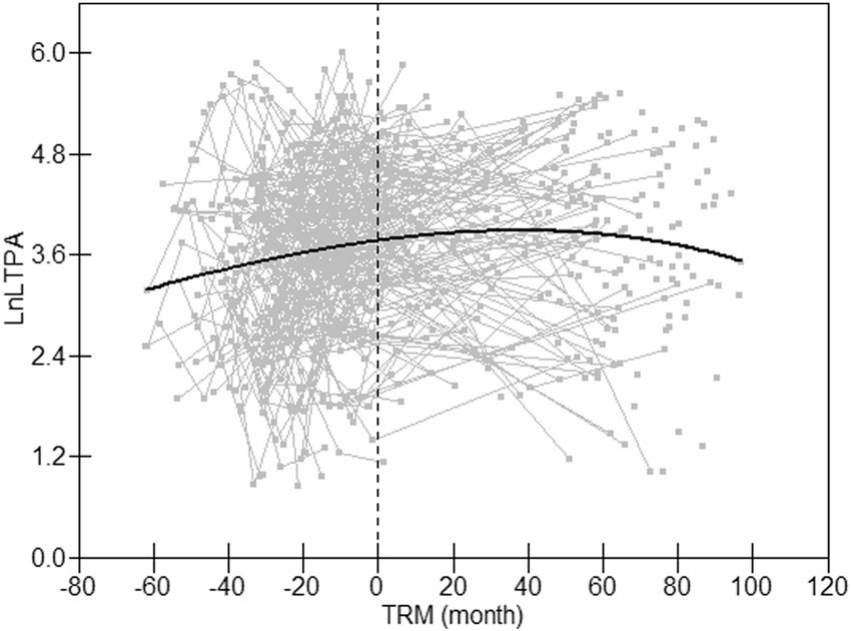
Longitudinal change pattern of LTPA. Data for LTPA are plotted against time relative to menarche (TRM). Gray lines represent longitudinal change of each individual’s value and the black line is the best fitting lines derived from hierarchical model.

Changes of amino acid levels during the 7.5-year follow-up are presented in Table 1. All amino acids, except alanine were higher at the age of 11 than at the age 18. LTPA increased gradually through puberty, peaked around 60 months after menarche and then decreased into early adulthood (Fig. 1). Body height, weight and BMI increased significantly over time (p < 0.05 for all).

**Table 1:** Physical characteristics and amino acids during growth.

	Baseline		7.5-year follow-up	
	Mean	95%CI	Mean	95%CI
Age(year)	11.2^a^	11.1–11.3	18.3	18.1–18.4
Height(cm)	145.6^a^	144.7–146.6	165.8	165.1–166.6
BMI	18.3^a^	18.0–18.7	21.9	21.5–22.3
Leucine (mmol/l)	0.076^a^	0.074–0.078	0.068	0.066–0.069
Isoleucine (mmol/l)	0.055^a^	0.053–0.056	0.044	0.043–0.045
Valine (mmol/l)	0.198^a^	0.193–0.203	0.169	0.165–0.173
Tyrosine (mmol/l)	0.061^a^	0.059–0.062	0.045	0.043–0.046
Phenylalanine (mmol/l)	0.071^a^	0.070–0.072	0.063	0.062–0.064
Glycine (mmol/l)	0.307^a^	0.301–0.314	0.251	0.244–0.257
Glutamine (mmol/l)	0.568^a^	0.558–0.578	0.520	0.512–0.529
Alanine (mmol/l)	0.342^a^	0.334–0.350	0.387	0.379–0.396
Histidine (mmol/l)	0.776^a^	0.752–0.780	0.553	0.538–0.567
LTPA (h/wk)	51.2	44.3–58.2	73.5	62.1–84.9

BMI = body mass index, LTPA = leisure time physical activity. Analysis of variance with repeated measurement was used to compare the differences between baseline and follow-up. a: compare with follow-up p < 0.05.

### Longitudinal associations between LTPA and amino acids

**Figure 2:**
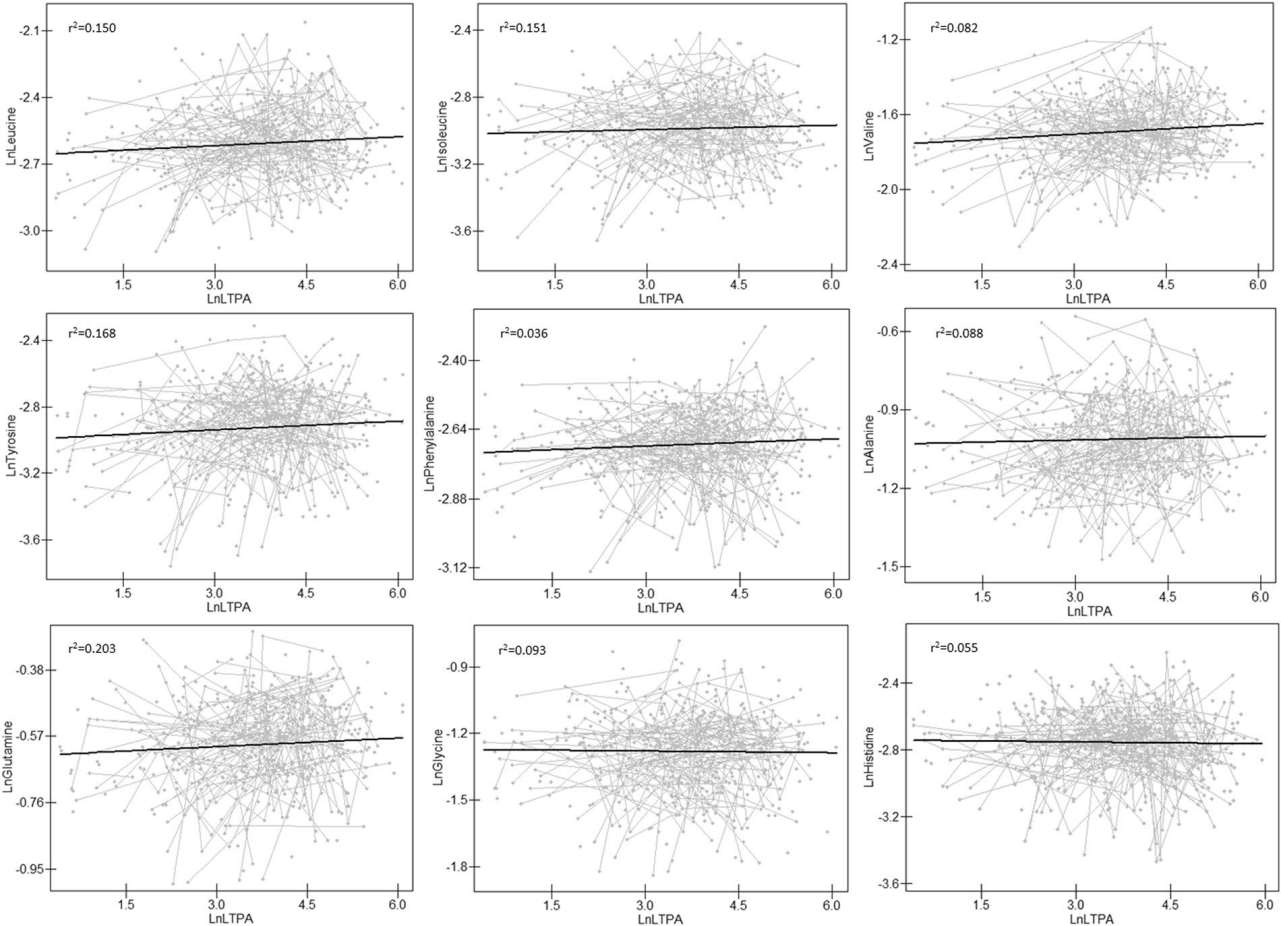
Longitudinal correlations between LTPA and amino acids. Data for amino acids (Y-axis) are plotted against LTPA (X-axis). Gray lines represent longitudinal change of each individual’s and the black lines are the best fitting lines derived from hierarchical models. The concentrations of amino acids and LTPA are back-transformed from natural log values, respectively.

Using hierarchical model, we found that LTPA correlated positively with leucine (r^2^ = 0.150, p < 0.01), isoleucine (r^2^ = 0.151, p < 0.01), valine (r^2^ = 0.082, p < 0.01), phenylalanine (r^2^ = 0.036, p < 0.05), tyrosine (r^2^ = 0.168, p < 0.05), glutamine (r^2^ = 0.203, p < 0.05), but not with alanine, glycine and histidine (Fig. 2). After adjusting for BMI, the association between LTPA and leucine, valine, phenylalanine, tyrosine, and glutamine remained significant.

**Figure 3:**
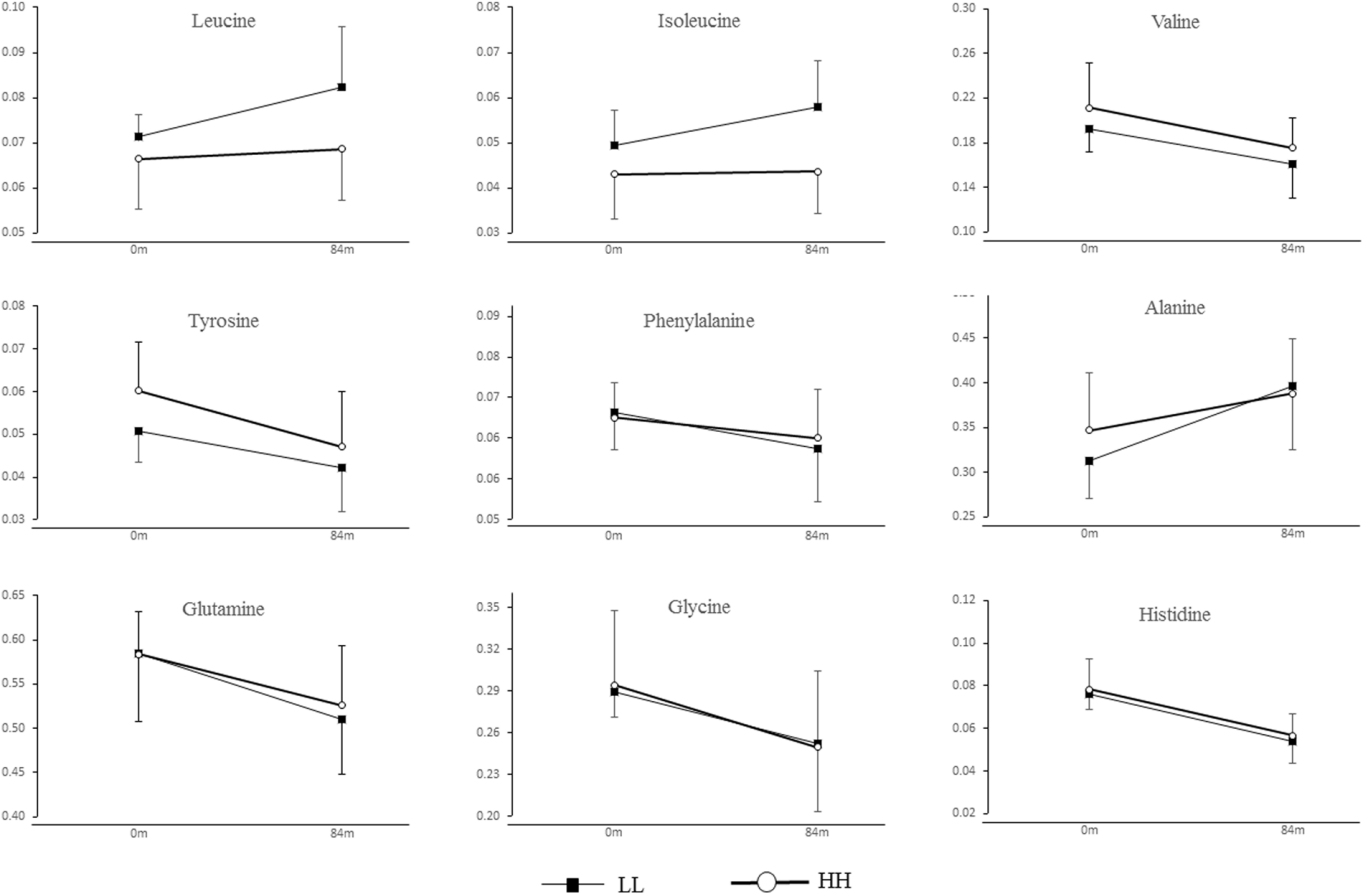
The change of amino acids between different consistently low active and consistently high active groups. The error bars represent the standard deviations. Analysis of variance with repeated measurement was used to compare the differences within and between groups.

We then assessed whether consistently different physical activity levels during the follow-up period associated with different amino acids concentrations. We found that isoleucine, leucine and tyrosine levels were significantly higher in individuals with lower LTPA than their peers at age 11 (p < 0.05 for all, Fig. 3). During the follow-up period, all amino acids changed significantly in both groups with directionally concordant effects (p < 0.01 for all). The only exceptions were isoleucine and leucine, which increased significantly (17.3% and 15.3%, respectively) among the individuals with consistently low LTPA, while among the individuals with consistently high LTPA the level of these amino acids remained virtually unchanged. Adjusting for baseline and follow-up BMI did not materially affect the results.

## Discussion

This longitudinal study from pre-puberty to early adulthood demonstrates that circulating amino acids are associated with LTPA during growth. Notably, we found that girls with consistently higher LTPA than their peers had significantly lower isoleucine, leucine and tyrosine levels at the age of 11. During the follow-up of 7.5 years, isoleucine and leucine levels increased significantly in girls with consistently low LTPA, while in girls with consistently high LTPA the level of these amino acids remained virtually unchanged.

Physical activity confers many health benefits, with higher physical activity levels associated with reduced risk of metabolic disorders such as obesity, insulin resistance and dyslipidemia. Physical inactivity and sedentary behaviors in turn are associated with metabolic inflexibility – an inability to switch between the oxidation of lipids and carbohydrates, which appears to be an important feature of cardio-metabolic disorders^12^. Recent studies suggest that increased branched-chain amino acid catabolism is intrinsically linked to increased fatty acid oxidation and better metabolic health in physically active individuals^6^, while decreased catabolism and increased circulating branched-chain amino acids are potential biomarkers for health and disease^13^, associated with impaired glucose and lipid metabolism in adolescents and young adults^14–16^. In the present study, we found that many amino acids were associated with LTPA during pubertal growth. Notably, girls with consistently high LTPA from age 11 to age 18 than their peers had significantly lower serum isoleucine and leucine levels. Furthermore, we found that in peripubertal girls with consistently low LTPA, isoleucine and leucine levels increased significantly (~ 15%), whereas no significant changes in these amino acids was found in girls with consistently high LTPA. This may reflect increased amino acid catabolism induced by higher level of physical activity. Interestingly, valine showed opposite tendency, though the differences were not statistically significant. The fact that valine decreased from baseline to follow-up in both consistently high and consistently low physical activity groups in a similar manner suggests that circulating valine concentrations are not largely affected by physical activity during pubertal growth. The reason for this is not clear, but it might be related to growth and sexual maturation as studies have demonstrated that adequate plasma valine levels is essential for sexual maturation in females^17,18^. Thus, it possible that circulating concentrations of valine maybe tightly defended during pubertal growth. These results suggest that that high level of habitual physical activity has the potential to favorably alter isoleucine and leucine levels during pubertal growth, while sedentary behavior appears to have opposite effects.

Direct comparison of our results with earlier studies is difficult because to our knowledge this is the first study investigating the longitudinal relationship between LTPA and circulating amino acids in children and adolescents. Overall, data on circulating amino acids levels in relation to habitual physical activity is limited, but there are few studies conducted in adults, which support our findings. Kujala *et al*. explored metabolomic signatures of habitual physical activity using twin pairs including males and females discordant for their leisure time physical activity for 30 years. Their results showed lower concentrations of serum isoleucine in the active versus inactive twin, and this finding was confirmed in three different population-based cohorts with age- and sex-matched pairs of unrelated adults identified as persistently active or persistently inactive^5^. These findings are supported by their earlier study, which showed that among the physically active members of twin pairs, compared with their inactive co-twins, genes related to branched-chain amino acid catabolism in muscle and adipose tissue were increased, together with increased expression of genes related to oxidative phosphorylation^4^. Recently Xiao *et al*. found lower levels of branched-chain amino acids among study participants with higher physical activity^19^. Similar findings were reported in in a population-based study of Japanese adults^20^.

The mechanism by which increased physical activity alters amino acid metabolism is not entirely clear, but it is likely to occur through increased amino acid oxidation during exercise. This seems plausible because amino acids, such as isoleucine and leucine, are oxidized largely in skeletal muscle, and exercise may activate enzymes responsible for the catabolism of these amino acids^21^. Animal studies support this hypothesis by showing that the prevention of branched-chain amino acid catabolism in mice by deleting the BCAT2 gene, which encodes the enzyme branched-chain aminotransferase that catalyzes the first reaction in the catabolic pathway causes exercise intolerance in mice, indicating that branched-chain amino acid catabolism is necessary for exercise performance^22^. Taken together, our results support the hypothesis that exercise and physical activity up-regulates the catabolic pathway of branched-chain amino acids^21^, which may serve as a mechanistic link between high level of physical activity and metabolic health in children and adolescents^23–25^.

Our study has some limitations that deserve mention. We used self-administered questionnaires to assess LTPA. While questionnaires have been reported to be the most feasible methods to estimate PA in large populations, they may not reflect the true level of PA because of possible over or under reporting^26^. Objective PA assessment with accelerometers or heart rate monitors may have provided more accurate estimates of PA, but given the relatively long follow-up period these methods were not feasible in our study. In addition, the study participants were all girls, and because there are distinct biological differences related to amino acid and protein metabolism between girls and boys during puberty, caution is advised if seeking to generalize from our results to boys. However, our study used unique longitudinal study design that spans from childhood across puberty to early adulthood in a representative sample of Finnish girls, thus we believe that this cohort is appropriate for studying the relationships between circulating amino acids and LTPA during growth from childhood to early adulthood. The strength of this study is also the rigor exhibited in collecting blood samples in a strictly defined period of the menstrual cycle in girls with regular menses.

In conclusion, high level of physical activity is associated lower serum isoleucine and leucine in peripubertal girls, independent of BMI, which may serve as a mechanistic link between high level of physical activity in childhood and its health benefits later in life. Further studies in peripubertal boys are needed to assess whether associations between physical activity and circulating amino acids in children adolescents are sex-specific.

## Materials and Methods

### Study Design and Subjects

The study cohort consisted of 396 girls who participated in a longitudinal study for, on average, 7.5 years with repeated measurements (Calex-study: determinants of body composition during growth). Detailed information regarding the participants has been reported previously^27^. Briefly, the subjects were first contacted via class teachers teaching grades 4 to 6 (age 10 to 12 year) in 61 schools in the city of Jyväskylä and its surroundings in Central Finland (96% of all the schools in these areas). 396 girls participated in the laboratory tests one to eight times over a maximum period of 8 years (mean duration of total follow-up was 7.5 years and mean age at last follow-up was 18.3 years). At the baseline, all girls were in Tanner stage I and II without menstruation. At the follow-up assessment, information regarding menstruation was collected by a questionnaire and phone calls. The age at menarche was defined as the first onset of menstrual bleeding. There were 13 girls who reported using oral contraceptives at the age of 18 years; these girls were excluded from the final analysis. Finally, for the purpose of this report, only girls with physical activity and serum amino acid measures were included (n = 221).

All information was collected and laboratory tests were performed within a two-week period during the same month throughout the 7.5-year follow-up to avoid seasonal effects. The study protocol was approved by the ethical committee of the Central Finland Health Care District. Written informed consent was obtained from all participants prior to the study. If the participant was underage, a written informed consent was obtained from parents or a legal guardian on behalf of their child. The study was conducted in accordance with the declaration of Helsinki.

### Anthropometry assessments

Body weight and height were measured with subjects wearing light clothes and without shoes. Weight was determined within 0.1 kg for each subject using an electronic scale and was calibrated before each measurement session. Height was determined using a fixed wall-scale measuring device to the nearest 0.1 cm. BMI was calculated use BMI = Weight (kg)/Height (m) ^2^.

### Physical activity assessment

LTPA level was evaluated using a self-administrated physical activity questionnaire described in our previous study^28^ and was a modified version of a validated questionnaire used in a previous WHO study^29^. Briefly, the questionnaire included questions about frequency and duration of physical activity, and what were the first, second, and third favorite physical activities subjects participated in outside of school. A LTPA score was calculated as: LTPA Score = frequency *∑1–3. Where frequency = times per week, 1–3 = the three physical activities, intensity index = MET value according to body mass, duration = hours per session, loading: non-weight bearing = 1 and weight bearing = 2. The LTPA scores for the girls were validated against activity energy expenditure estimated from doubly-labeled water and indirect calorimetry when girls were at the age of 18(n = 17, r = 0.651) and was reported in our previous paper^28^.

### Blood sample analysis

Blood samples were collected in the morning between 7:00 and 9:00 a.m. after overnight fasting at each time point. The blood samples were collected 2 to 5 days after menstruation among those girls with regular menses. Serum was extracted by centrifugation and stored immediately at −80 °C until analyzed.

### Serum amino acids assessments

Serum amino acids were analysed using a high-throughput serum NMR metabolomics platform; the experimental protocols including sample preparation and NMR spectroscopy have been described in detail elsewhere^30–32^. The metabolomics platform that was used to assess circulating amino acids only provides data on 9 amino acids. Nine amino acids (alanine, glutamine, glycine, histidine, isoleucine, leucine, phenylalanine, tyrosine and valine) were quantified in millimoles per liter. CV% for alanine, glutamine, glycine, histidine, valine, leucine, isoleucine, phenylalanine, and tyrosine were 2.6%, 3.2%, 3.5%, 4.15, 2.7%, 3.6%, 4.8%, 3.9%, and 5.3%, respectively. To address how well leucine was resolved from its isomer isoleucine, leucine signal was located at 0.974 ppm (i.e. 487.3 Hz at 500 MHz NMR spectrometer). The isoleucine signal was located at 1.022 ppm (i.e. 511.3 Hz). The typical line width at half height for leucine and isoleucine signals is circa 1.5 Hz. Thus, isoleucine and leucine signals were clearly resolved.

We focused mainly on branched-chain amino acids their association with health and disease which is widely acknowledged, and because they were the only amino acids in our study that showed conspicuously different patterns in relation to consistently high and consistently low physical activity.

### Statistical analysis

All data were checked for normality using the Shapiro-Wilk’s *W*-test in IBM SPSS Statistics 22.0 for Windows. If data were not normally distributed, their natural logarithms were used. Descriptive statistics were presented as means and 95% confidence interval (CI) for the mean at the three follow-ups. Data of the different time points were compared each other using the general linear model.

A hierarchical nonlinear model with random effects was employed to assess the associations between the changes in LTPA and metabolite concentrations from pre-puberty to early adulthood (MLwiN 2.20 software, Multiple Project, Institute of Education, University of London, UK). The hierarchical model allows inclusion of the data from every subject despite irregularity of temporally spaced follow-up or missing data^33^. Furthermore, to test if consistency of physical activity level during adolescence had significant effects on serum amino acid levels girls divided into two groups according their LTPA scores by median values then four groups were formed as described previously^34^. For this report, only consistently high active (5.5 → 5.1 hours/week, 5.5 → 4.2 times/week n = 50) and consistently low active (2.2 → 2.2 hours/week, 2.3 → 1.6 times/week n = 53) groups were included.

Analysis of variance with repeated measurement was used to compare the differences between the baseline and follow-up in Table 1 and the differences within and between groups in Fig. 3.
